# Endometriosis-Related Spontaneous Hemoperitoneum in the Third Trimester: A Case Report

**DOI:** 10.7759/cureus.32897

**Published:** 2022-12-24

**Authors:** João Pedro Gomes Pereira, Carolina Vaz-de-Macedo, Mariana Almeida, Luis Canelas

**Affiliations:** 1 Department of Obstetrics and Gynecology, Hospital Garcia de Orta, Almada, PRT

**Keywords:** rupture, pregnancy, uterine artery, hemoperitoneum, endometriosis

## Abstract

Spontaneous hemoperitoneum in pregnancy (SHiP) is a rare but significant condition in pregnancy and is linked to high rates of morbidity and mortality. Endometriosis increases the risk of SHiP, particularly during the third trimester of pregnancy. We report a case of a 45-year-old woman in the third trimester of a pregnancy complicated by SHiP due to the rupture of a uterine artery by an endometriosis implant, which is a particularly rare cause.

## Introduction

Endometriosis is a common gynecologic disorder that affects approximately 10% of reproductive-aged women. It is a chronic inflammatory condition defined by the presence of endometrial-like tissue outside of the uterine cavity [1]. Spontaneous hemoperitoneum in pregnancy (SHiP), a potentially fatal intra-abdominal bleeding, is an uncommon but serious complication of endometriosis during gestation [2]. Due to the limitations of non-invasive techniques, endometriosis diagnosis without surgical investigation is challenging [3]. As SHiP is a potentially life-threatening condition, health care professionals should be aware of it in women with a history of endometriosis.

## Case presentation

A 45-year-old woman, gravida 2 para 0, with history of deep endometriosis treated with laparoscopy five years before, presented to the emergency department at 35 weeks of gestation due to severe abdominal pain. Her pregnancy had been uneventful until the week before this episode, when she had been admitted to the hospital for four days due to hemoperitoneum in the context of decidualization of endometriotic lesions. She had been discharged hemodynamically stable with clinical improvement.

Physical examination revealed a pregnant abdomen consistent with 35 weeks, and abdominal guarding with rebound tenderness. The uterus was extremely tender, and the cervix was long and closed. Obstetric ultrasound showed a viable singleton pregnancy with a normally implanted placenta and hydramnios. The patient was hemodynamically stable (blood pressure of 109/55 mmHg and pulse of 82 bpm), and blood tests revealed a hemoglobin level of 11.0 g/dL, normal coagulation tests, and a negative C-reactive protein level.

**Figure 1:**
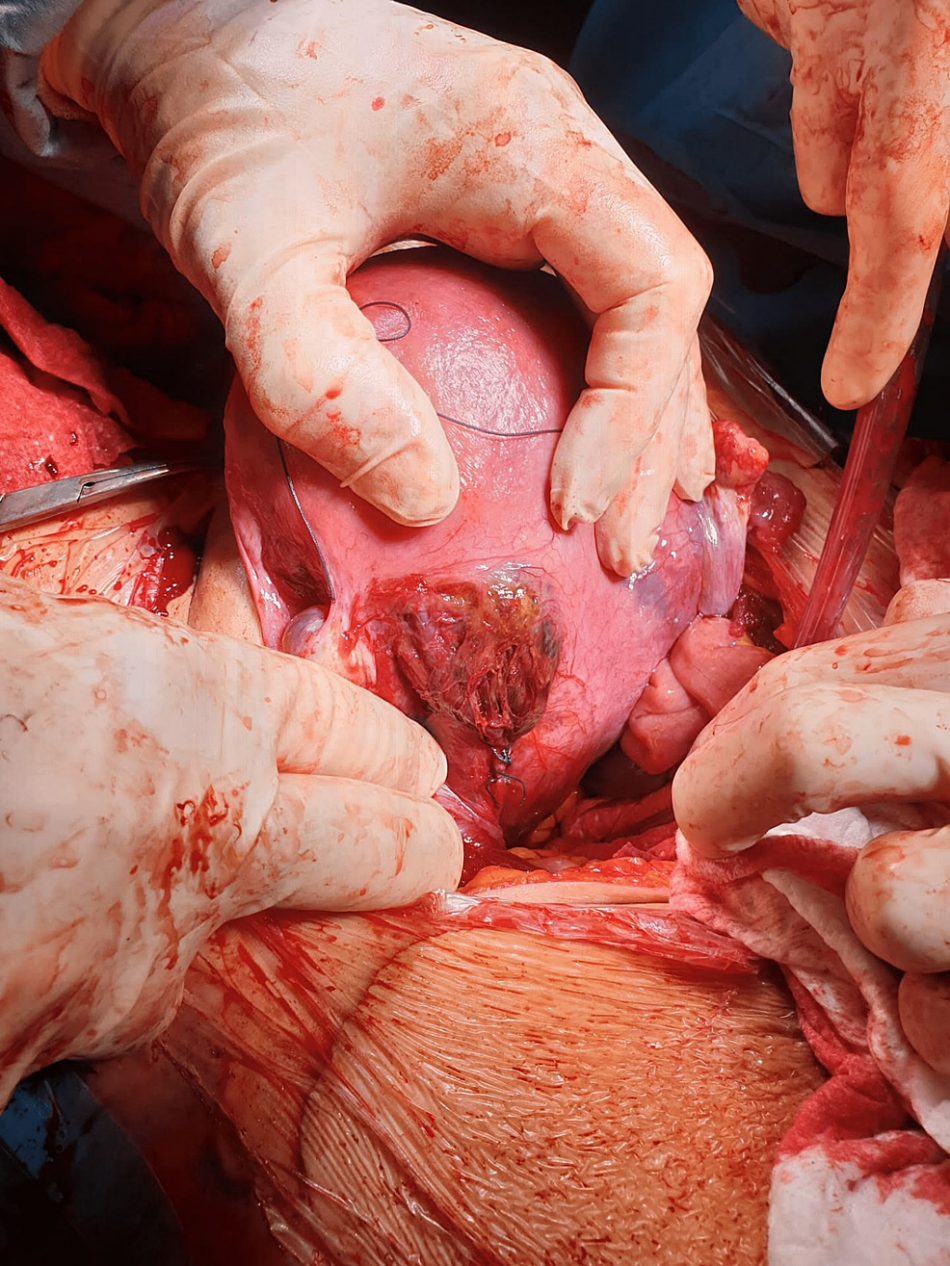
Uterine artery ripped and stretched by a suspected endometriotic adhesion

Due to sustained fetal bradycardia detected on cardiotocography, an emergency lower-segment transverse cesarean section was performed under general anesthesia. A baby weighing 2,730 grams was delivered with Apgar scores of 2, 8, and 8 at 1, 5, and 10 minutes, respectively. The peritoneal cavity was aspirated, yielding more than 2,000 mL of blood. Active bleeding was discovered from fragile tissue over the left lower portion of the uterine posterior surface following the evacuation of the hemoperitoneum. A presumed endometriotic adhesion tore and strained the left uterine artery at the posterior uterine wall (Figure 1). Hemostasis was achieved with multiple hemostatic stitches on the uterine wall, and an examination of the abdominal cavity revealed no other sources of bleeding. The patient received two units of fresh frozen plasma and two units of red blood cells intraoperatively. The patient’s immediate postoperative period was complicated by an aspiration pneumonia, and the patient was discharged seven days after the delivery, clinically improved. Three months later, she reported no sequels.

## Discussion

This report illustrates a case of SHiP, a rare complication of endometriosis, which presented with an acute abdomen and fetal distress after rupture of the left uterine artery. SHiP occurs most frequently during the third trimester of pregnancy. Ginsburg et al. [4] reported that 32% of out-of-labor SHiP cases occurred at term, 39% occurred between 33 and 37 weeks of gestation, and 29% occurred before 33 weeks of gestation. Similar to our case, 89.5% of SHiP cases presented as acute or subacute abdominal pain. Clinical examination may raise suspicion of hemoperitoneum in virtually all patients with a sharp decline in hemoglobin, symptoms of hypovolemic shock, or an altered trace on cardiotocography [5].

SHiP is mostly associated with the venous system. However, in very rare cases as this one, arterial ruptures have been described as the origin of bleeding. The second stage of labor, muscular activity, coughing, defecating, or other physical exertion can all increase the venous pressure in the utero-ovarian circulation, which is already increased during pregnancy [2]. Brichant et al. [5] described a case of spontaneous uterine artery rupture in association with endometriosis in a 41-week pregnant woman who had been admitted for induction of labor. Due to evidence of fetal distress on cardiotocography and hypovolemic shock, she underwent emergency cesarean, during which the surgeons found active bleeding from the right uterine artery in the area of the parametrium. As in our case, successful hemostasis was achieved after ligation of the uterine artery. To the best of our knowledge, this is the first report of SHiP after left uterine artery rupture in association with endometriosis.

The idea that endometriosis might contribute to the etiology of SHiP was initially proposed in 1992 [6]. According to this theory, the association between endometriosis and SHiP would occur due to a variety of pathophysiological processes, including the following: (1) blood vessels increased vulnerability to rupture due to the endometriosis chronic inflammation, (2) pelvic adhesions and an enlarged uterus would enhance the risk of vessel rupture, [7] and (3) first-trimester decidualization, which causes endometriosis lesions to enlarge during pregnancy and become resistant to progesterone, may also play a role in the development of SHiP [5]. The observation that most SHiP bleeding locations are, as in our case, on the left hemipelvis, the side where endometriotic implants are predominantly found [8], supports the hypothesis that endometriosis and SHiP are related. Despite accumulating evidence that endometriosis is a causal factor in the development of SHiP, it is currently impossible to identify which patients are at a particular risk of developing the condition [8].

A correlation between severity of SHiP and the stage of endometriosis has not been established, and several aspects of endometriosis treatment are still up for debate. It is unknown if surgical endometriosis therapy prevents pregnancy issues such as SHiP in addition to improving fertility [2]. Additionally, extensive surgery may have detrimental effects by leading to the formation of adhesions and further impairment of delicate intra-abdominal tissues [8]. Our patient had had a previously laparoscopy for deep endometriosis, which can be a risk factor for SHiP.

Because there may be obstetric and surgical diseases that could be confused with SHiP and require rapid surgical intervention, the correct diagnosis of SHiP is rarely made prior to surgery [9]. Laparotomy and emergent cesarean are often necessary to manage these patients [2]. Perinatal morbidity/mortality remains a serious problem for SHiP, which does not seem to have been altered over the past few decades [10]. Interestingly, although it would be reasonable to expect that cases derived from uterine artery rupture could have a particularly poor outcome, in both our case and the previously reported one [5], management by prompt surgical intervention was successful. As previously mentioned, we think that the key to better results is enhanced awareness to enable prompt recognition and diagnosis of SHiP [5].

## Conclusions

Uterine artery rupture is a particularly rare cause of SHiP, for which endometriosis is a risk factor. This is an unusual but serious pregnancy complication that is linked to poor pregnancy outcomes. Perinatal mortality and morbidity of SHiP remain high despite overall improved medical care during pregnancy. Health care practitioners should be aware of this entity as a potential differential diagnosis as a timely diagnosis could significantly enhance maternal and fetal outcomes.
